# Regeneration Therapy: The Role of Platelet-Rich Plasma in Post-hysterectomy Wound Dehiscence Healing

**DOI:** 10.7759/cureus.48062

**Published:** 2023-10-31

**Authors:** Shreya A Sahu, Deepti Shrivastava

**Affiliations:** 1 Obstetrics and Gynaecology, Jawaharlal Nehru Medical College, Datta Meghe Institute of Medical Sciences, Wardha, IND

**Keywords:** hysterectomy, wound infections, autologous platelet-rich plasma, wounds and healing, platelet-rich plasma (prp)

## Abstract

A 58-year-old (para 2, living 2, abortion 0), overweight (BMI: 25 kg/m2), post-hysterectomy patient reported with wound dehiscence on day seven. She was a known case of hypertension and type 2 diabetes for the last five years. She presented with symptoms of abnormal uterine bleeding due to leiomyoma. The leiomyoma was refractory to medical management and thus she underwent a total abdominal hysterectomy. She underwent the procedure well after preoperative intensive diabetes and hypertension management. She was managed postoperatively with injectable antibiotics. On day seven postoperatively, the patient started experiencing wound discharge, after which she was treated with broad spectrum higher antibiotics and regular wound dressing with debridement of necrotic debris twice daily for five days. She was planned for alternative therapy in the form of rejuvenation therapy by platelet-rich plasma therapy, which thus helped further shorten her hospital stay and helped the wound to heal better.

## Introduction

Hysterectomy is the second most common operative procedure performed on women in the world after lower segment caesarean section. Worldwide, its incidence varies between 6.1 to 8.6 per 1000 women of all ages. The most common indications are abnormal uterine bleeding (AUB) associated with fibroid, endometriosis, adenomyosis, and uterovaginal prolapse. Post-hysterectomy scar dehiscence occurs around 0.6% of the time worldwide [1]. A lower segment caesarean section, a traditional caesarean section, or prior uterine trauma would be typical significant factors [2,3]. The triad of ileus, vomiting, and coughing, as well as obesity, pre-existing pulmonary and cardiovascular conditions, vertical incisions, and, to a lesser extent, hypoproteinaemia, electrolyte and fluid imbalance, and wound infection, are the variables that are related to the risk of wound dehiscence [4,5]. The frequency of abdominal wound dehiscence would be significantly reduced if high-risk patients were recognised, proper wound toilet was employed, sepsis was rapidly treated with abdominal decompression, and close attention was paid to electrolyte and protein balance in the pre- and postoperative period [6]. In this regard, platelet-rich plasma (PRP) therapy is a novel technique, as it infiltrates the wound site with growth factors, thereby allowing for better recovery and wound healing [7].

## Case presentation

A case of a 58-year-old, post-menopausal (para 2, living 2, abortion 0), overweight (BMI: 25 kg/m2) woman presented with AUB. Her ultrasonograph showed ill-defined, heterogenous fibroids in the posterior wall of the uterus with posterior myometrial thickness being 11.2 mm. She was a known case of controlled hypertension, hypothyroidism, and type 2 diabetes for the last five years. Her case of leiomyomas was refractory to medical management, and so she was planned for definitive management with a total abdominal hysterectomy. She underwent the procedure well after preoperative intensive diabetes and hypertension as well as hypothyroidism management. She was managed postoperatively with injectable antibiotics and regular insulin.

On day seven postoperatively, the patient started experiencing wound discharge. The patient first presented with tachycardia with temperature spikes of about 101-103°F. The patient's wound was gaping and leaking pus. The abdominal results and the per vaginal examination were also unremarkable but for the wound infection. Investigations of the patient revealed elevated white blood cell counts (20,100/mm3) along with normal haemoglobin (10.2 gm%) and platelets (3.18x105/mm3). The rest of her biochemistry measurements were normal.

It was decided to use meropenem 1 g twice a day (BD) as the preferred medication to treat the wound infection. It was also followed up with regular wound dressing with debridement twice daily for five days. As the wound did not show significant signs of improvement, and given the case had signs of advanced age with various comorbidities like type 2 diabetes mellitus, hypertension, and hypothyroidism, the need for management that could hasten her healing process was dire. With the patient's permission, PRP therapy was started at this time due to the severity of several comorbidities, the length and expense of the wound's re-suturing, as well as the wound's failure to respond to normal therapy.

She was given regular wound care and PRP treatment (the dose was 3-5 ml once per day/alternate days until the wound was healed). For this type of wound, she was scheduled for rejuvenation therapy using leukoreduced PRP (LR-PRP) therapy. At the point of care, PRP was readily created. The patient's entire blood was centrifuged twice, once to separate the plasma from packed red blood cells and once to separate platelet-poor plasma from PRP (in PRP-approved tubes, which are categorized as class II equipment). Lab grade 15 cc centrifuge tubes were used. We took two 15 ml tubes and added 1.5 ml anticoagulant to each tube and 13.5 ml blood was collected by using scalp vein set 18 number directly to the anticoagulant-added tubes. A ratio of 1 ml anticoagulant to 9 ml blood was maintained. Later, the tubes were tightly capped and mixed well by inversion several times. It was then centrifuged for 15 minutes at 2000 rpm followed by which plasma rich in platelets was separated. At least 50% volume was plasma on top and there was no buffy coat on the red blood cells layer.

Then, plasma was transferred to another tube using a sterile pipette without picking red blood cells. It was centrifuged at 3500 rpm for 15 minutes to get a platelet pellet, which left behind 10% plasma (10% of the original blood collected) above the pellet. Lastly, the platelet pellet was re-suspended in the remaining plasma using a sterile pipette, and syringes were loaded and used immediately. Complete blood count (CBC) prior to PRP preparation to determine baseline platelet count and leukocyte count was as follows: haemoglobin = 10.8 gm%, total leukocyte count = 6900/mm3, platelets = 3.8x105/mm3. We kept a sample aside and ran CBC again to check for platelet count, and the PRP value was at least five times the baseline value. Also, the leukocyte count (as we were using LR-PRP) was higher compared to the baseline. As the parameters were met, we injected freshly made LR-PRP. It was planned to be injected in the wound every alternate day for a duration of a few weeks till significantly visible wound healing was noticed. The wound was dressed with Vaseline-covered gauze pieces to prevent it from getting absorbed or escaping the dressing.

**Figure 1:**
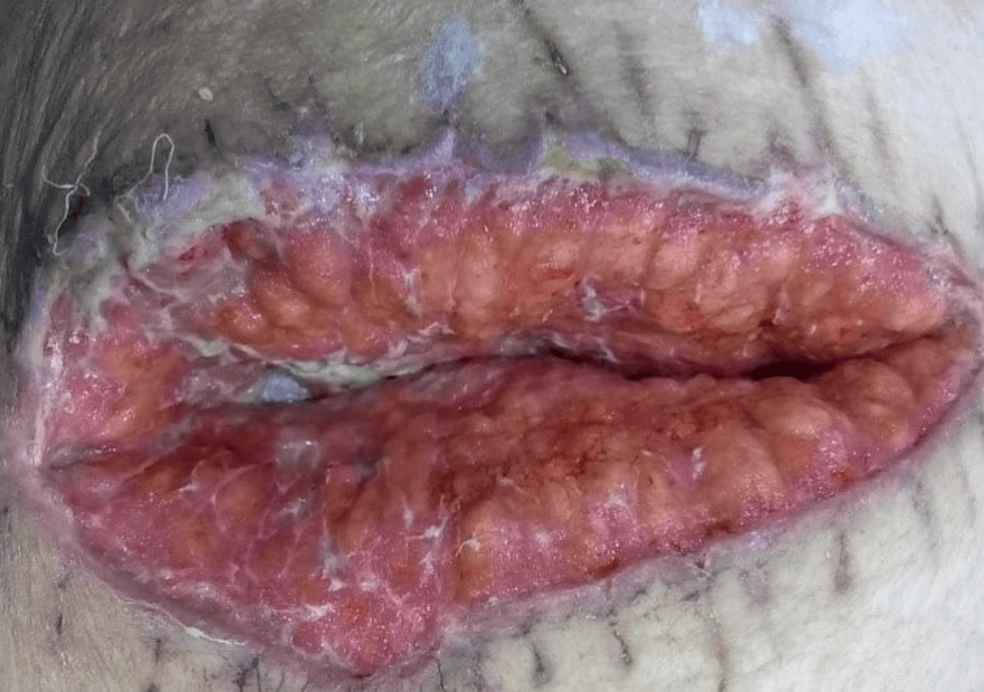
The wound was examined and found to be 10 cm long, 4 cm wide, and 3 cm deep (wound depth measured by finger). The wound had minimal fibrin deposition, was not epithelized, and was hyperpigmented. Granulation tissue was evident and was firm and deep pink. The wound borders were curled, and the peri-wound area was erythematous.

**Figure 2:**
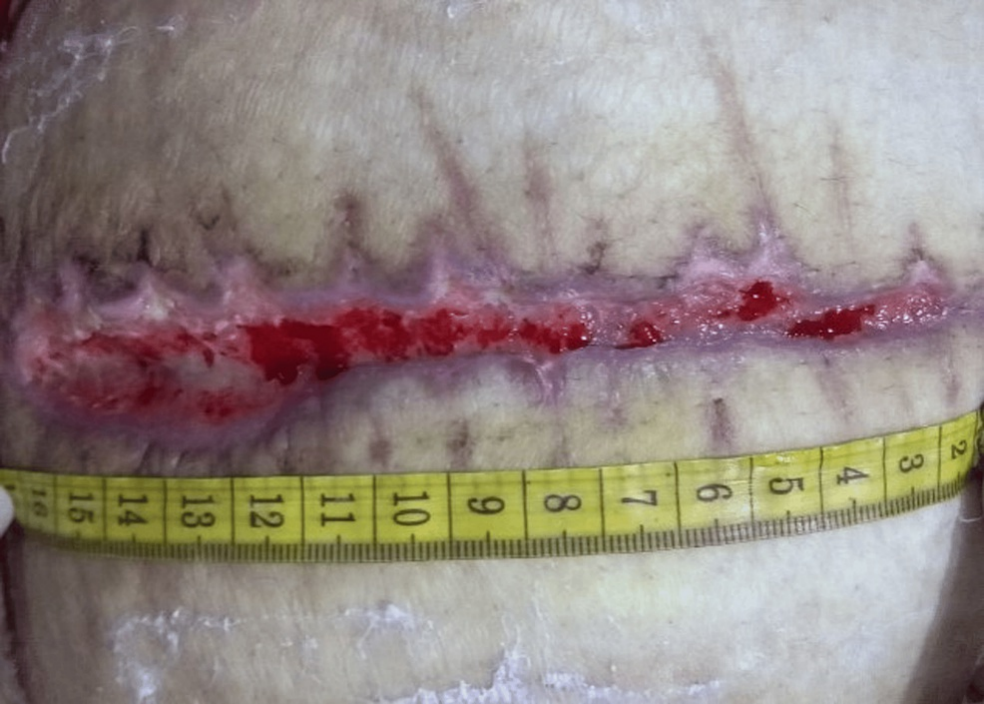
The wound was noticeably shallower in week two of platelet-rich plasma therapy; however, it had gotten better over the past two weeks. It had a considerable fibrin accumulation and was still only around 50% epithelial. The wound borders were now intact and there was firm, pink granulation tissue, indicating regenerating epithelium.

**Figure 3:**
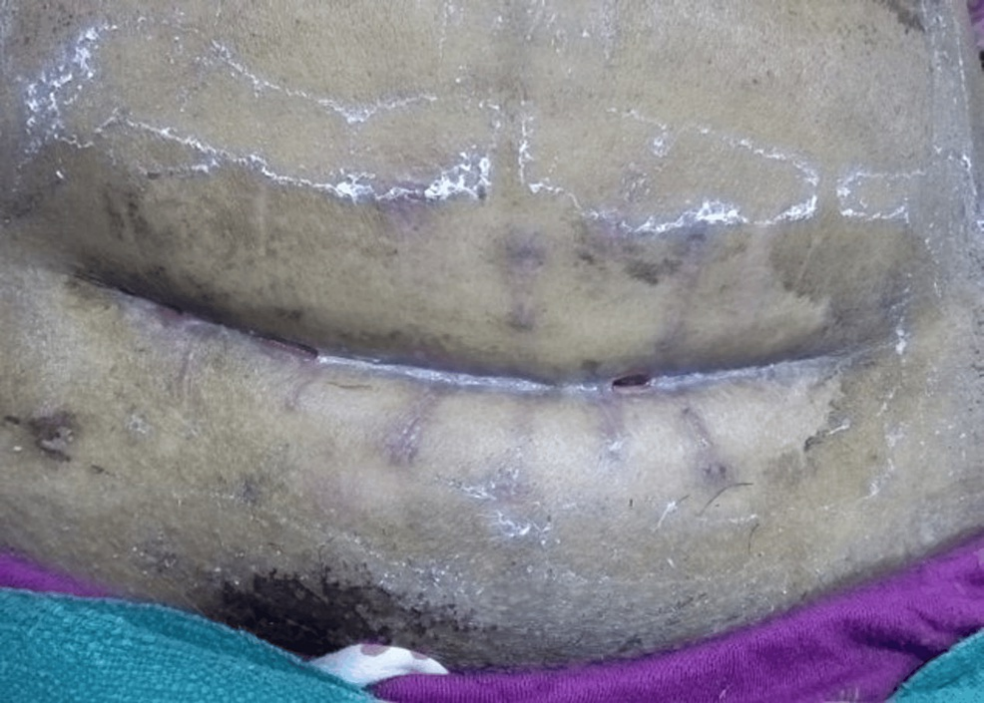
After three weeks of platelet-rich plasma therapy, the wound had significantly improved. Measuring 10 cm long and free of tunnelling, the wound was obviously smaller in all directions. It had significant fibrin accumulation and was entirely epithelial. The edges were undamaged.

Progression of wound healing was quantified as per the standard hospital protocol based on the following parameters: visible contractility, epithelialization, amount of fibrin deposition, and granulation tissue formation. A track of these parameters of wound healing progression was kept until the wound was completely healed, which took three weeks. The results of the same have been depicted in Figures 1-3. This procedure of PRP instillation and regular aseptic wound care and dressings proved successful in this case and the patient was continued on injectable antibiotics for five days, followed by oral antibiotics thereafter for two weeks. The patient was also counselled about routine hygiene maintenance along with a high protein diet and exogenous protein supplementation was also prescribed for three weeks to help speed up the healing process. It reduced her length of stay in the hospital and accelerated the healing of the incision. A continuous reduction in wound depth after a few weeks of PRP treatment was accompanied by observable changes in the length and breadth of the wound. The wound contracted in all directions after three weeks of wound care visits, with a markedly reduced depth.

## Discussion

The present case shows the effectiveness of PRP therapy in managing the abdominal wound dehiscence following hysterectomy, a surgical complication, thereby averting several postoperative morbidities and patient and doctor discontent. In this particular case, one of the circumstances that may have influenced wound dehiscence was obesity with pre-existing comorbidities, some fluid and electrolyte imbalance, and wound infection [7,8]. Autologous PRP, which is produced as needed from the patient's blood, provides the growth factors for wound healing and gaping. Moreover, it is much safer due to its autologous nature and is proven to be an effective active intervention for this type of wound [9]. Moreover, the additional advantages of PRP include the treatment of infections by attracting white blood cells. As a result, PRP treatment was shown to be the least intrusive, least stressful, and most affordable choice. This led to a shorter hospital stay and no additional surgical intervention [10].

The findings were in line with the study by Tehranian et al. [11], where patients undergoing lower caesarean sections had significantly lower pain scores and scar scores with better healing of the caesarean wound. However, its efficacy is being continuously explored not only for hysterectomy or caesarean wounds but a number of other types of wounds as well [12] because the efficacy of PRP may depend on a variety of factors like preparation method, concentration of platelets, single vs. double spin method, storage conditions, application experience, and topical vs. injectable nature of administration [13].

The present case overall reflects the effectiveness of PRP in post-hysterectomy wound healing, allowing for better recovery and management of the patient. However, the study was limited by a short follow-up, without any blinding of the study procedure.

## Conclusions

It can be concluded that PRP is a novel method for wound healing for the patient in the post-hysterectomy period. The autologous nature of the PRP makes it of minimal danger to the patient in terms of associated complications. However, future trials are needed to compare its use against other wound healing methods. Moreover, specific guidelines need to be standardized for the preparation and application of PRP for various types of wounds.

## References

[1] Diaz SD, Jones JE, Seryakov M, Mann WJ (2002). Uterine rupture and dehiscence: ten-year review and case-control study. South Med J.

[2] Wagner MS, Bédard MJ (2006). Postpartum uterine wound dehiscence: a case report. J Obstet Gynaecol Can.

[3] Metgud MC, Kataria A, Nadipally SR, Patil K (2020). Incidence of wound dehiscence following obstetric and gynecological surgeries at a tertiary care hospital: a retrospective study. J South Asian Feder Obst Gynae.

[4] Helmkamp BF (1977). Abdominal wound dehiscence. Am J Obstet Gynecol.

[5] Mowat J, Bonnar J (1971). Abdominal wound dehiscence after caesarean section. Br Med J.

[6] Guo S, Dipietro LA (2010). Factors affecting wound healing. J Dent Res.

[7] Dhurat R, Sukesh M (2014). Principles and methods of preparation of platelet-rich plasma: a review and author's perspective. J Cutan Aesthet Surg.

[8] Hussein IH, Zalikha AK, Tuluca A, Crespi Z, El-Othmani MM (2022). Epidemiology of obese patients undergoing revision total knee arthroplasty: understanding demographics, comorbidities, and propensity weighted analysis of inpatient outcomes. J Am Acad Orthop Surg Glob Res Rev.

[9] Lacci KM, Dardik A (2010). Platelet-rich plasma: support for its use in wound healing. Yale J Biol Med.

[10] Stanirowski P, Sawicki W (2013). Modern methods of therapy of hard-to-heal post-operative wounds in obstetrics and gynecology - analysis of applicability and effectiveness of use. Postępy Nauk Medycznych.

[11] Tehranian A, Esfehani-Mehr B, Pirjani R, Rezaei N, Sadat Heidary S, Sepidarkish M (2016). Application of autologous platelet-rich plasma (PRP) on wound healing after caesarean section in high-risk patients. Iran Red Crescent Med J.

[12] Xu P, Wu Y, Zhou L (2020). Platelet-rich plasma accelerates skin wound healing by promoting re-epithelialization. Burns Trauma.

[13] Park YB, Kim JH, Ha CW, Lee DH (2021). Clinical efficacy of platelet-rich plasma injection and its association with growth factors in the treatment of mild to moderate knee osteoarthritis: a randomized double-blind controlled clinical trial as compared with hyaluronic acid. Am J Sports Med.

